# Radially patterned transplantable biodegradable scaffolds as topographically defined contact guidance platforms for accelerating bone regeneration

**DOI:** 10.1186/s13036-021-00263-8

**Published:** 2021-03-22

**Authors:** Yonghyun Gwon, Sunho Park, Woochan Kim, Taeseong Han, Hyoseong Kim, Jangho Kim

**Affiliations:** 1 Department of Rural and Biosystems Engineering, Chonnam National University, Gwangju, 61186 Republic of Korea; 2 Interdisciplinary Program in IT-Bio Convergence System, Chonnam National University, Gwangju, 61186 Republic of Korea

**Keywords:** Critical-sized bone defect, Bone tissue engineering, Radially pattern, Transplantable, Biodegradable scaffold

## Abstract

**Background:**

The healing of large critical-sized bone defects remains a clinical challenge in modern orthopedic medicine. The current gold standard for treating critical-sized bone defects is autologous bone graft; however, it has critical limitations. Bone tissue engineering has been proposed as a viable alternative, not only for replacing the current standard treatment, but also for producing complete regeneration of bone tissue without complex surgical treatments or tissue transplantation. In this study, we proposed a transplantable radially patterned scaffold for bone regeneration that was defined by capillary force lithography technology using biodegradable polycaprolactone polymer.

**Results:**

The radially patterned transplantable biodegradable scaffolds had a radial structure aligned in a central direction. The radially aligned pattern significantly promoted the recruitment of host cells and migration of osteoblasts into the defect site. Furthermore, the transplantable scaffolds promoted regeneration of critical-sized bone defects by inducing cell migration and differentiation.

**Conclusions:**

Our findings demonstrated that topographically defined radially patterned transplantable biodegradable scaffolds may have great potential for clinical application of bone tissue regeneration.

## Background

Treatment of large critical-sized bone defects remains a major clinical orthopedic challenge. Critical-sized bone defects are technically defined as those that will not heal spontaneously during a patient’s lifetime [1, 2]. These defects may occur after acute trauma, chronic cases of nonunion, or oncological cases following resection [3, 4]. These critical-sized bone defects may negatively impact an individual’s life, making it crucial that patients receive rapid and complete treatment. The current gold standard for treating critical-sized bone defects is an autogenous bone graft. However, autogenous bone grafts are associated with several critical limitations, including the high cost of surgery, requirement of complicated microsurgery techniques, donor site morbidity, and limited treatment success of large bone defects [5–10].

Bone tissue engineering (BTE) aims to facilitate and accelerate the body’s natural healing capacity using scaffolds produced from engineered biomaterials. An effective BTE scaffold is required in order to achieve the ultimate goal of bone regeneration. The following functional characteristics of BTE scaffolds are indispensable for their successful clinical application: (1) excellent biocompatibility and biodegradable, (2) enhancement of cellular phenomena, such as proliferation, migration, adhesion, and differentiation, (3) ability to provide sufficient space for the growth of cells, (4) suitable mechanical properties, including flexibility, and (5) easy handling during surgery [11–13]. Accordingly, BTE scaffolds for successful clinical application focuses on the use of biocompatible biodegradable polymer-based biomaterials. Polymer-based BTE scaffolds can be broadly classified into the following: natural polymers, such as collagen, alginate, and chitosan; and synthetic polymers, such as polycaprolactone (PCL), poly-lactic-co-glycolic acid (PLGA), and polylactic acid (PLA) [14–18]. Among natural polymers, collagen-based scaffolds, such as Geistlich Bio-Gide® membrane, are the most commonly used in the clinical field [19, 20]. However, it is well known that collagen-based scaffolds have many critical limitations, including high swelling and bioresorption rates, low adhesive and mechanical properties, and high costs [21, 22]. In the current study, we selected PCL, a synthetic polymer that is able to overcome the above noted limitations. PCL is commonly used as a BTE scaffold due to its characteristic strengths, such as its biodegradability, biocompatibility, high rigidity, and flexibility. In addition, PCL is a common material used to manufacture US Food and Drug Administration (FDA)-approved devices and is relatively inexpensive [23–26]. Therefore, PCL is considered to be an ideal candidate for producing transplantable biodegradable scaffolds for use in bone regeneration.

Living cells are highly sensitive to the complex, but well-defined structural extracellular matrix (ECM) environment that can regulate cell fate and function [27, 28]. The ECM has various topographical characteristics that range from a microscale to nanoscale. For example, bones consist of collagen and hydroxyapatite, and the ECM of bone tissue are arranged in a complex hierarchical structure with microscale grooves [11, 29]. The behavior of host cells and osteoblasts in bone tissue is dependent on the structure and orientation of the topographical cues. In this respect, topographical guidance has been continuously studied as a strategy to enhance cellular phenomena, such as proliferation, migration, and differentiation, and also to promote tissue regeneration [30, 31]. In particular, many studies have been published regarding the regeneration of various tissues using micro-nano scale aligned topography; the strategies involved include the control of cell morphology and cellular behavior [27, 32–34]. However, research regarding radial topography in the control of cellular behavior and enhancing tissue regeneration is very limited. Yoon et al., reported a surface with radial topography that promotes cell migration and bone regeneration [35]. However, this study had certain limitations in that it used materials that were non-biodegradable and had low biocompatibility for clinical BTE application.

In the current study, we proposed a topographically defined transplantable scaffold composed of a biodegradable polymer—capable of recruiting host cells and guiding their migration into bone defects—as a treatment modality for critical-sized bone defects. For the execution of this concept, we designed a PCL-based scaffold with centrally aligned radial microgrooves. First, the mechanical properties of three different types of scaffolds (flat, aligned, and radial) were analyzed and compared to evaluate differences in mechanical strength based on their topographical structure. Second, their effects on cell proliferation, migration, and differentiation were investigated in vitro. Finally, in vivo critical-sized bone defect regeneration was performed using a commercially available collagen-based product (currently the most commonly used scaffold worldwide) and compared at 4 wk. and 6 wk. post-treatment*.*

## Materials and methods

### Design and fabrication of the radially patterned PCL-based scaffold

Radially patterned PCL scaffolds imprinted directly onto silicon wafers are not easily peeled off due to the surface charge of silicon wafers. Therefore, we used a polydimethylsiloxane (PDMS) mold. First, the PDMS pre-polymer Sylgard 184 Silicon Elastomer (Dow Corning) was mixed with 10% curing agent, poured into a petri dish to a sufficient thickness, and baked at 70 °C for at least 2 h to ensure curing without any residue. The polymer was then peeled off from the petri dish and oxidized for 5 min and 15 min at a wavelength 100 nm using an UV treatment system. To ensure uniform oxidation, the distance of the polymer from the UV lamp was constant at 5 cm. The UV/ozone dose was 15 mW/cm^2^ (measured at a distance of 10 mm at wavelengths of 185 nm and 254 nm). Thereafter, 100 mL of 10% (w/v) adhesion promoter 3-(trimethoxysilyl) propyl methacrylate (TMSPMA) was drop-dispensed onto the UV/ozone-treated PDMS sheet. To replicate radial structures (5 μm wide and deep) on PDMS, a polyurethane acrylate (PUA) precursor (Changsung Sheet, Korea) was drop-dispensed and UV-cured for use as a self-replicating mold. Finally, additional UV-curing was performed for more than 10 h to remove any non-cured acrylate groups. The UV-assisted PUA precursor solution was dropped onto the silicon master mold, which was then covered with 100-μm thick poly (ethyleneterephthalate) film (SKC, Korea). For curing, the master mold covered with the PUA precursor was exposed to UV light (λ = 310–400 nm, 40 W) for 30 s [11].

PCL beads (Mw: 80,000; Sigma Aldrich, USA) were dissolved in dichloromethane (18% wt.) by mixing for 1 d using a magnetic stirrer. To fabricate the PCL scaffold, PCL solution was poured onto a clean square piece of glass (18 mm × 18 mm) and spin-coated at 3500 rpm for 240 s. The glass coated with PCL was then pressed at ~ 70 kPa with flat PDMS fabricated on a hot plate at 80 °C. Following the imprinting process, the fabricated flat PCL scaffolds were cooled to room temperature and the PDMS mold peeled from the PCL scaffolds. Using the same process, the aligned and radial PCL scaffolds were pressed at ~ 70 kPa with aligned and radial patterned PDMS, respectively, that were fabricated on a hot plate at 70 °C. Likewise, the aligned and radial patterned scaffolds were cooled to room temperature and the PDMS mold peeled from the aligned and radial PCL scaffolds.

### Scaffold characteristics and properties analysis

Field emission scanning electron microscopy (FE-SEM) images of the surfaces of various patterned scaffolds fabricated in the study were captured using a JSM7500F FE-SEM microscope (Oxford, UK) at an acceleration voltage of 15.0 kV and average working distance of 8.8 mm. The samples were coated with platinum prior to morphological observation. Chemical characteristics of the flat, aligned, and radial scaffolds were analyzed to confirm their chemical variations. The chemical bond structures were examined by Fourier-transform infrared spectroscopy (FT-IR) using a FT-IR spectrometer (Spectrum 400, USA).

The mechanical strain and stress tests of the various patterned PCL scaffolds were performed using MCT-1150 tensile testers (A&D Company, Japan) at a test speed of 100 mm/min. The tests included the analysis of 10 specimens per sample with the same interval set. Normal and shear adhesion forces of various patterned PCL scaffolds were evaluated using porcine rind and measured using an MCT-1150 instrument at a test speed of 50 mm/min. Prior to the adhesion test, a fresh porcine rind was rinsed with deionized water, the various patterned PCL scaffolds were attached to its surface, and rind/scaffolds measured under a preload of ~ 0.5/cm^2^. The pulling weight was gradually increased until the adhesion force felled off.

### Immunofluorescence

Adherent cells on various patterned PCL scaffolds were fixed with a 4% paraformaldehyde solution (Biosesang, Korea) for 15 min and permeabilized with 0.2% Triton X-100 (Biosesang). The fixed cells were then blocked with 3% normal goat serum (NGS; Abcam, Cambridge, MA, USA) in phosphate-buffered saline (PBS; Biosesang). After being washed with PBS, the scaffolds were stained with tetramethylrhodamine (TRITC)-conjugated phalloidin (Millipore) for 1 h and then with 4, 6-diamidino-2-phenylindole (DAPI; Millipore) for 3 min. Images of the stained cells were obtained using a fluorescence microscope. For quantitative analysis of the morphology of the MG-63 cells on the substrates, images were obtained using a fluorescence microscope and analyzed using Image J software.

### Cell attachment and proliferation analysis

We quantified the cell attachment using the WST-1 assay, which is the same method as previous studies [33, 36]. Specifically, Osteoblast-like MG-63 cells (1 × 104 cells/samples) were seeded onto the scaffold samples and cultured in Dulbecco’s Modified Eagle Medium (DMEM) containing 10% fetal bovine serum (FBS) and 1% antibiotics (Cellgro, USA) for 6 h, 3 d and 5 d at 37 °C in a humidified atmosphere containing 5% CO2. Quantitative analysis of the proliferation of cells growing on the samples was performed by WST-1 assay using a Premix WST-1 Cell Proliferation Assay System (Takara Bio Inc., Kusatsu, Japan). To confirm cell attachment prior to performing the cell proliferation assays, the scaffolds were washed with PBS to remove any non-adherent cells.

### Osteogenic mineralization analysis

Osteoblast-like MG-63 cells (4 × 10^4^ cells/sample) were cultured for 7 d or 14 d on scaffold samples in osteogenic differentiation medium (100 nM dexamethasone, 50 μm ascorbic acid, and 10 mm glycerol 2-phosphate in DMEM). After 7 d, the cells were washed with PBS and fixed in 4% paraformaldehyde for 15 min. The fixed cells were stained with Alkaline Phosphatase Blue Membrane Solution (Sigma Aldrich, USA) to confirm early osteogenic differentiation of the osteoblasts on the scaffold surfaces. The stained cells were then de-stained using a SensoLyte pNPP Alkaline Phosphatase Assay Kit (AnaSpec, Inc., USA) and the extracted stain then measured using a microplate reader at 405 nm to quantify the osteogenic differentiation of the osteoblasts. In addition, MG-63 cells were stained with Alizarin Red S (Sigma Aldrich, USA) after 14 d of culturing to confirm later osteogenic differentiation according to the degree of mineralization of the MG-63 cells on sample surfaces. To quantify the osteogenic differentiation of the MG-63 cells, the Alizarin Red S stained cells were then de-stained with cetylpyridinium chloride (Sigma Aldrich) and the extracted stains quantitated using an iMarkTM Microplate Absorbance Reader (Bio-Rad, Hercules, CA, USA) at 595 nm.

### In vivo animal study

The animal study was approved by the Ethics Committee of Chonnam National University. Six-week-old male C57Bl/6 N mice were assigned to six different groups (*n* = 5/group): Defect (control), flat, aligned, radial, and collagen-based products (Geistlich Bio-Gide; Geistlich Pharma, Switzerland). The mice were fully anesthetized with an intraperitoneal injection of Zoletil (0.006 cc/10 g) and Rumpun (0.004 cc/10 g). The heads of the mice were then shaved and disinfected. The bones were exposed by incising the skin approximately 3.0 cm above the calvaria bone. Bone defects (diameter: 5 mm) were generated on the one side of the exposed calvaria bone using an electric drill. Prepared scaffolds (Diameter: 5 mm) were placed on the calvarial bone defect. After suturing the skin, the ambient temperature was raised and the mice were woken from the anesthesia. The mice were euthanized 4 wk. or 6 wk. after surgery and tissues obtained, including the calvaria bone and defect region. Calvaria bone microcomputed tomography (μ-CT) was performed using a Skyscan 1172 Micro-CT (Skyscan, Konitch, Belgium) at a resolution of 11.38 pixels and exposure time of 316 ms, with an energy source of 80 kV and current of 124 μA. An average of 488 slices of calvaria bone were scanned. The μ-CT images were analyzed using MIMICS 14.0 3D imaging software (Materialise’s Interactive Medical Image Control System, Leuven, Belgium). The calvaria bone specimens were fixed in 10% formalin and decalcified in 0.5 M ethylenediaminetetraacetic acid (pH 7.4) at room temperature for 7 d. After the specimens were embedded in paraffin, they were cut into 5-mm thick sections and then stained with hematoxylin and eosin (H&E). Images were obtained using Aperio ImagesScope software (Leica, CA, USA).

### Migration assay

A thin PDMS slab was used to provide a cell-free area for investigating the migration and wound-healing effects of osteoblasts on the samples. Specifically, a 5 mm thick PDMS sheet was cut into 5 mm round slabs using a steel punch. The PDMS slabs were placed onto the scaffold surfaces. MG-63 cells (1 × 10^5^ cells/samples) were cultured on the wound-generated samples. The PDMS slabs were then manually removed using sharp tweezers and the cells of the wound were evaluated by immunofluorescence 24 h post-culturing using a fluorescence microscope (Zeiss, Germany). The cell-covered area and cell migration rate in the wound were evaluated using Image J.

### Western blotting

For western blot analysis, RIPA Cell Lysis Buffer (Biosesang) containing Xpert Proteinase Inhibitor Cocktail (GenDEPOT, Houston, TX, USA) was used to lyse cells. Protein from the lysed cells was incubated on ice for 30 min and then centrifuged at 13,000 rpm for 30 min at 4 °C. Protein concentrations were quantitated using a DC Protein Assay Kit (Bio-Rad, Hercules, CA, USA) and the Lowry assay. Equal amounts of proteins were loaded onto sodium dodecyl sulfate polyacrylamide gel electrophoresis gels, the proteins were resolved by electrophoresis, and then transferred onto polyvinylidene difluoride membranes (IPVH00010; Millipore) using an electroblotting apparatus at a constant voltage of 30 V for 1 h. The membranes were blocked for 1 h with 5% skim milk in Tris-buffered saline containing Tween 20 (TBST). Subsequently, the membrane was incubated with the following primary antibodies overnight at 4 °C: extracellular signal-related kinase (ERK, 1:1000; Santa Cruz Biotechnology, CA, USA), focal adhesion kinase (FAK, 1:1000; Cell Signaling Technology, Beverly, MA, USA), and glyceraldehyde 3-phosphate dehydrogenase (GAPDH, 1:5000; Abcam). Goat anti-mouse IgG (H + L)-HRP and goat anti-rabbit IgG (H + L)-HRP (GenDEPOT, Houston, TX, USA) were used as secondary antibodies at a dilution of 1:10,000 to probe the membrane for 1 h at room temperature. The membranes were then washed three times for 10 min each in TBST. Bands were detected using West Pico PLUS Chemiluminescent Substrate (Thermo Scientific, Waltham, MA, USA).

### Growth factors assay

To ascertain whether the MG-63 cells could secrete growth factors and cytokines on the radially surface, the cells (3 × 10^4^ cells/samples) were seeded on the substrates and cultured for 5 days in the proliferation medium. The cells were then cultured for 3 days in the osteogenic differentiation medium. Finally, the cells were again cultured for 1 day in DMEM. using the RayBio G-Series Human Growth Factor Array 1 Kit (RayBiotech, Norcross, GA) according to the manufacturer’s protocol. The assay kit was first treated with blocking buffer for 30 min. Then the culture medium containing the secreted proteins was incubated with the kit for 2 h at room temperature. After washing with wash buffer, a biotinylated antibody cocktail was added for 2 h, following which the kits were washed several times. Cy3 equivalent dye-conjugated streptavidin was then incubated with the kit for 1 h, following which washing was carried out several times. Fluorescence images of the reacted kits were obtained using the GenePix 4000A (Molecular Devices).

### Statistical analysis

All quantitative data are presented as the mean ± standard deviation. Unpaired Student *t*-tests were used for statistically analyzing the results of cell adhesion, viability, and differentiation experiments. To compare three or more conditions, one-way ANOVA was performed. *P*-values less than 0.05 were considered significant. Micro-CT scans were subjected to the Kruskal-Wallis test for statistical analyses using the SPSS software.

## Results

### Design and characteristics of the PCL flat, aligned and radial patterned scaffolds

**Fig. 1:**
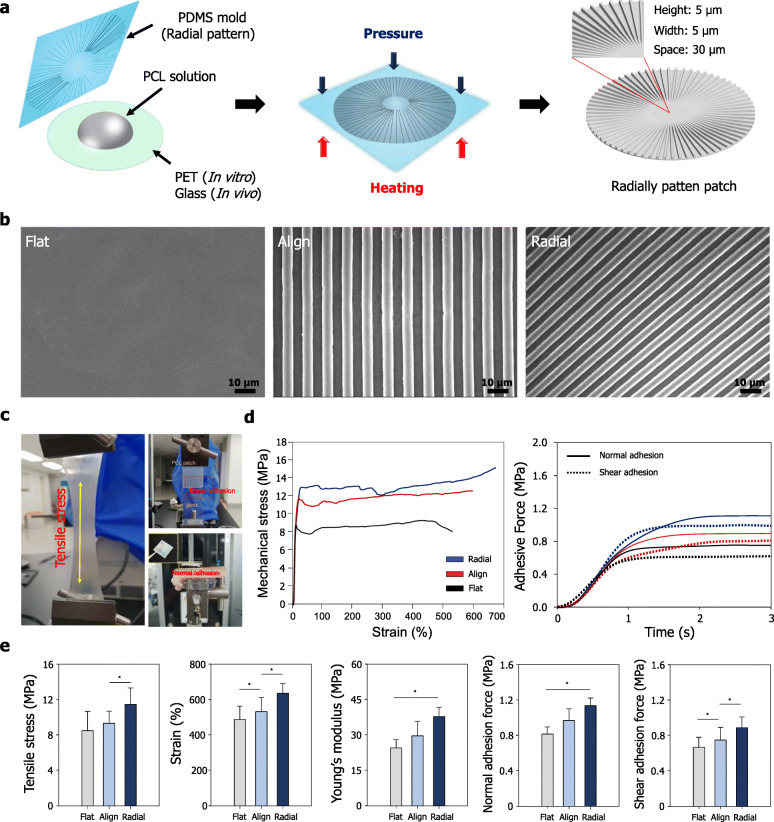
Fabrication and characteristics of the topographically defined PCL-based scaffold. **a** Schematic of the fabrication methodology used to generate the radial topography PCL scaffold using lithography technology. **b** FE-SEM images of the flat, aligned, and radial topography surfaces of the PCL scaffolds. **c** Images of the mechanical properties test of the three types of PCL-based scaffold. **d** Strain-stress curves and adhesion force curves of the three types of PCL-based scaffold. **e** Quantitative analysis of tensile stress, strain, Young’s modulus, normal adhesion force, and shear adhesion force of the three types of PCL-based scaffold (*N* = 10)

A schematic of the fabrication process of the radial patterned PCL scaffolds using lithography technology used in this study is shown in Fig. 1a. The FE-SEM images of the surface morphology of the three different types of scaffolds (flat, aligned, and radial) revealed different topographical orientations. The align and radial patterned scaffolds show the aligned topography along the pattern direction compared to flat patterned scaffolds. As shown in the Fig. 1b, the diameter of the pattern and the spacing between patterns are equal to 5 μm in align and radial pattern; however, the direction of the two patterns is different. Specifically, the radial scaffold showed a align pattern direction toward the center. To confirm the maintenance of the chemical properties of the three types of scaffolds, functional groups of the scaffolds were evaluated by FT-IR spectroscopy (Supporting Fig. 1). The characteristic absorption bands related to PCL (CH_2_ asymmetric stretching at 2944 cm^− 1^, symmetric stretching at 2866 cm^− 1^, C=O stretching vibration of carbonyl groups at 1721 cm^− 1^, and deformation of C–O at 1161 cm^− 1^) were detected for all scaffolds.

To confirm the mechanical properties of the three types of fabricated scaffolds, we investigated the effect of different topographies on the mechanical properties of the scaffolds by calculating the strain, stress, and Young’s modulus from stress–strain curves (Fig. 1c and d). The tensile strength and strain of the radial patterned scaffolds (~ 11.6 MPa and 630%, respectively) were higher than those of the aligned patterned scaffolds (~ 9.8 MPa and 560%, respectively) and flat patterned scaffolds (~ 8.1 MPa and 490%, respectively). Moreover, the Young’s modulus value for the flat and aligned patterned scaffolds was 23.2 and 31.2 MPa, respectively, whereas that for the radial patterned scaffolds was 38.4 MPa (Fig. 1e).

To analyze the effects of different topographies on the tissue-bonding performance of the scaffolds, we compared the normal and shear adhesive forces among the three scaffold types (Fig. 1c). As shown in Fig. 1d and e, the radial patterned scaffolds exhibited higher normal adhesive force (114 kPa) than that of the aligned patterned scaffolds (92 kPa) and the flat patterned scaffolds (81 kPa). Similarly, the radial patterned scaffolds displayed higher shear (85 kPa) and adhesive forces compared to those of the aligned patterned scaffolds (66 kPa) and flat patterned scaffolds (59 kPa). On the basis of these results, we demonstrated the mechanical properties of radially patterned PCL scaffolds are improved than those of previously reported patch-type non-patterned and fibrous PCL-based scaffolds [37–39].

### In vitro analysis of cell behavior on flat, aligned, and radial patterned PCL scaffolds

**Fig. 2:**
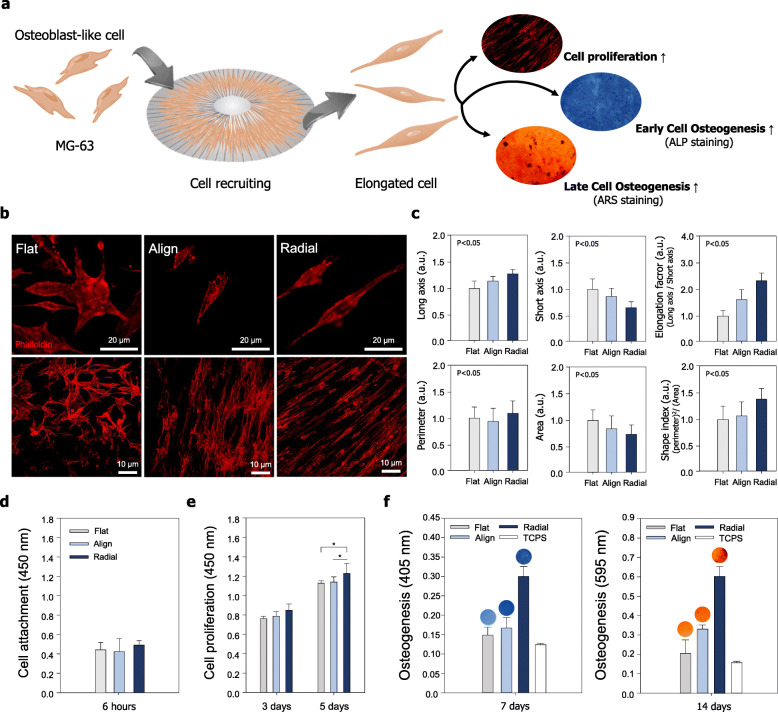
Effect of topographically defined surfaces on cellular behavior. **a** Schematic of the effect of radial topography on cell morphology, proliferation, and differentiation. **b** Immunofluorescence image at the multi-cell and single-cell levels of phalloidin (red)-stained osteoblast-like cells cultured on the three types of PCL-based scaffold. **c** Quantitative analysis of the cell body (*N* = 30). **d**-**e** Quantitative analysis of cell attachment and proliferation on the three types of PCL-based scaffold (*N* = 6). **f** Quantitative analysis of alkaline phosphatase staining of osteoblast-like cells during osteogenesis induction on the three types of PCL-based scaffold (*N* = 6). **g** Mineralization (ARS) staining of osteoblasts during osteogenesis on the three types of PCL-based scaffold (*N* = 6)

It is well known that topographical structure of the scaffolds affects cellular morphology and function [27, 28, 40, 41]. Immunofluorescence was used to investigate the effects of the different topographical cues on shape and orientation at a single-cell level. As shown in Fig. 2a, the radial topographical cue greatly influenced cell polarity, as indicated by the aligned cytoskeletal structure of cells. Cells on grown on structures with radial topography were more elongated compared to those grown on structures with aligned and flat topographies (Fig. 2a). The cells grown on structures with flat topography (un-patterned surface) showed relatively spherical shapes compared to those grown on structures with aligned and radial topographies (Fig. 2b). Cell elongation factors (CEFs), defined as (major axis)/(minor axis), were calculated to quantify the observed elongation of cells. Surprisingly, the CEF of cells grown on radial topography was approximately twice greater than that of cells grown on flat topography (Fig. 2c). Meanwhile, the area of cell body spread decreased on the radial topography (Fig. 2c). Here, we introduced the cell shape index (CSI), which was calculated as follows: CSI = (cell perimeter)^2^/(cell area). With the increasing trend of CEF, higher CSI values observed on the radial topography, indicating the cell body may be closely interconnected to each other in order to regulate their functions [11].

To investigate whether different topographies influenced cell proliferation and cell attachment, we cultured MG-63 cells on the three types of scaffolds for 6 h (cell attachment assay), 3 d (cell proliferation assay), and 5 d (cell proliferation assay) (Fig. 2d and e). After 6 h of culture, unattached cells were removed by washing with PBS and adhered cells on the scaffolds were quantified by WST-1 assays. We determined MG-63 cells were well attached on all scaffolds, irrespective of topographic properties. However, cells grown on the radial patterned scaffolds showed higher attachment than those of the aligned patterned scaffolds and flat patterned scaffolds. After 3 d and 5 d of culture, cell proliferation was greater on the radial patterned scaffolds compared with those of the other two scaffolds (Fig. 2e).

Phosphatase deposition of osteoblast-like cells on the three scaffold types was also examined by culturing MG-63 cells on the scaffolds in osteogenic induction medium for 7 d. Alkaline phosphatase staining revealed higher osteogenic levels on the radial patterned scaffolds compared to that on the aligned patterned scaffolds, the flat patterned scaffolds, and the tissue culture polystyrene substrate (TCPS). Osteogenic mineralization of MG-63 cells on the three types of scaffolds was also examined by culturing the cells on the scaffolds in osteogenic induction medium for 14 d. Alizarin Red S staining (Fig. 2f) revealed higher calcium expression levels on the radial patterned scaffolds compared to that on the aligned patterned scaffolds, the flat patterned scaffolds, and the TCPS. The alkaline phosphatase staining (early osteogenic differentiation) and Alizarin Red S staining (late osteogenic differentiation) demonstrated improved bone differentiation in the radial patterned scaffolds compared to that observed in the other two scaffolds.

### Bone tissue regeneration in vivo

**Fig. 3:**
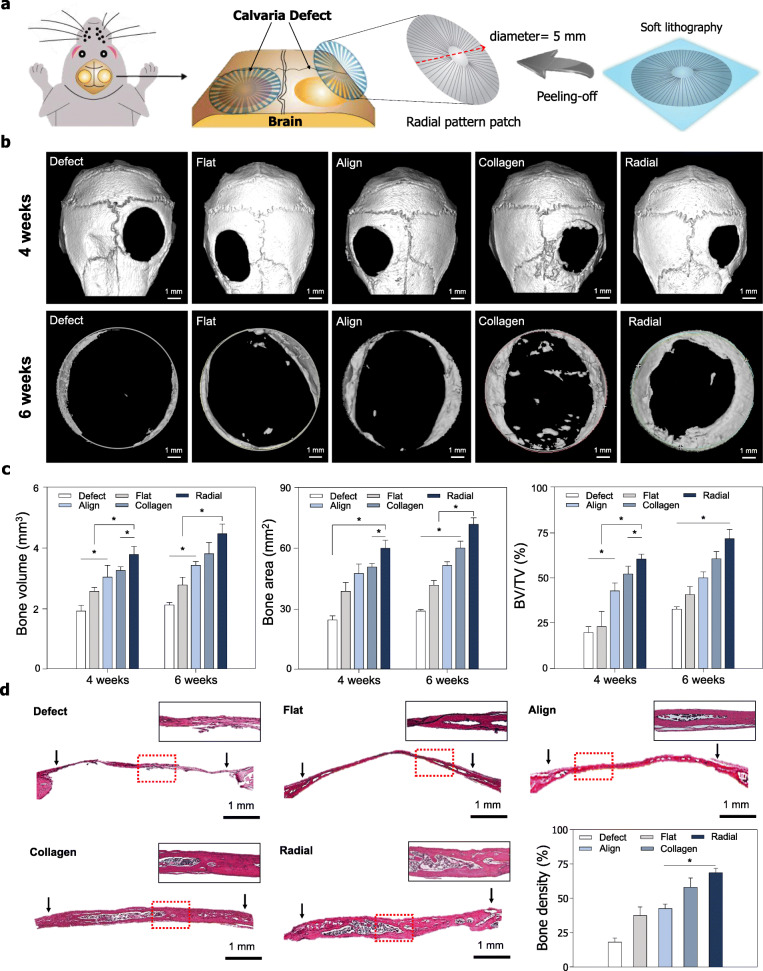
Effect of micro-topographical cues in vivo. **a** Schematic of the effect of radial topography on regeneration of critical-sized bone defect. **b** Representative micro-CT image. **c** Quantitative analysis of micro-CT imaging of bone regeneration after 4 wk. and 6 wk. of regenerative repair (*n* = 5 per sample). **d** Micrograph of hematoxylin and eosin (H&E) staining and quantitative analysis of bone density using the H&E images

All mice used in the in vivo studies survived till the euthanization date and no adverse reactions were observed. The three scaffold types (5 mm diameter) and collagen-based commercial products were placed on the surgically induced bone defect. No infection or inflammatory reaction was observed in any of the mice at any point during the postoperative period. The in vivo bone regeneration results confirmed the effects of radial topography (Fig. 3a). The scaffolds persisted for 6 wk. without deformation. Quantitative assessment of the effects of radial topography on bone formation was performed on new bone defects in vivo using micro-CT and 3D-image conversion with the MIMICS 14.0 software. As shown in the 3D images (Fig. 3b), bone formation in the radial topography group occurred along the periphery of the bone defect and regeneration occurred along the direction of pattern. Interestingly, after 4 wk. and 6 wk., compact bone formation was not observed in the defect group or flat group of mice, whereas bone regeneration was significantly enhanced in the radial patterned scaffold group after 4 wk. and 6 wk. of implantation compared to that in the commercial products group. New bone formation was observed from the edge to center, depending on the radial direction. At 6 wk., the bone volume was 2.11 mm^3^ in the defect group, 2.67 mm^3^ in the flat patterned scaffolds group, 3.52 mm^3^ in the aligned scaffold group, 3.85 mm^3^ in the commercial products group, and 4.45 mm^3^ in the radial patterned scaffolds group (Fig. 3c). The bone area was 29.81 mm^2^ in the defect group, 39.78 mm^2^ in the flat patterned scaffold group, 52.12 mm^3^ in the aligned scaffold group, 59.98 mm^3^ in the commercial product group, and 71.55 mm^3^ in the radial patterned scaffold group (Fig. 3c).

To analyze bone regeneration efficacy from radial topography, we performed H&E staining 6 wk. after implantation (Fig. 3d). The histological analysis also confirmed greater bone formation—with a dense cytoplasm—in the radial patterned scaffolds compared to that in the other pattern, even the commercial products scaffolds. These results regarding bone regeneration and formation provide insights into the importance of radial topographical cues for inducing bone tissue regeneration.

### Radial topographical cues on cellular migration and growth factor secretion

**Fig. 4:**
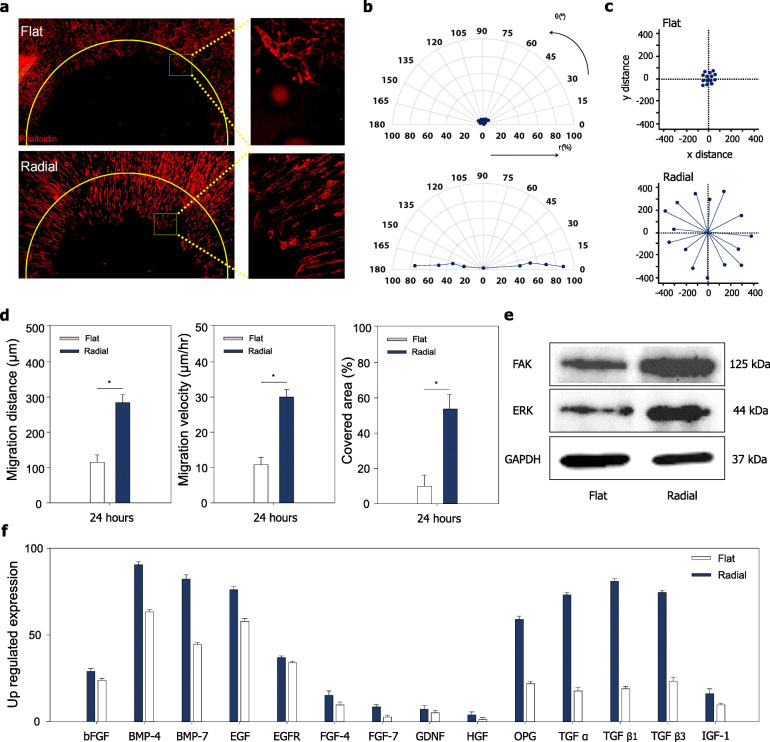
Alignment of cells on micro-topographically defined PCL-based scaffolds during in vitro cellular migration. **a** F-actin staining of migrating cells (phalloidin staining, red). The right image shows an enlarged image of the area in the yellow square. Scale bars = 100 μm. **b** Quantitative analysis of the cell alignment by determining the angles between the F-actin-stained cells and the direction of microgrooves, *r* = Percentage of cell orientation (%) / θ = Angle between F-actin and micropattern (°) (**c**) Cell migration distances on the flat and radial topographical surfaces after 24 h (*N* = 15). **d** Quantitative analysis of cell migration distance, velocity, and covered area (*N* = 15). **e** Western blot analysis and quantification of FAK and ERK expression levels in osteoblasts cultured on the flat and radial topographical surfaces for 2 d. **f** Quantification of the protein expression of the growth factor and cytokine arrays (*N* = 3)

To explore a possible mechanism on the enhanced bone regeneration in the radial topographical cues, we investigated interaction between the cells and radial topographical scaffolds in terms of cellular migration and growth factor secretion. To generate a cell-free area on the PCL scaffolds for the cell migration assays, PDMS sheets (5 mm in diameter) were placed as a mask at the center of the PCL scaffolds (Fig. 4a). We then mimicked the in vivo process of bone repair in the calvaria defects using the in vitro migration assays. After MG-63 cells were seeded and the PDMS sheets removed, cells were allowed to migrate toward the empty area at the center of the PCL scaffolds for 24 h. The orientation of the MG-63 cells at 24 h was then evaluated by phalloidin staining (Fig. 4a). Interestingly, the cells on the radial patterned PCL scaffolds exhibited aligned morphologies arranged along the orientation of the radial topography. In contrast, there was no alignment of the cells on the flat PCL scaffolds. Quantification of the cell alignment on the radial topography showed that 85% of the cells were affected by topographical cues and aligned along the orientation of the radial topography (Fig. 4b). Quantification of cell migration was also analyzed for the flat and radial topographies (Fig. 4c). The results showed enhanced migratory behaviors of cells grown on the radial topography compared to those grown on the flat topography. The migration distance (280 μm) and migration velocity (29 μm/h) of cells grown on the radial topography were higher than those of cells grown on the flat topography (115 μm and 12 μm/h, respectively) (Fig. 4d). Similarly, the cell-free area was determined after cell migration for 24 h. Migrating cells covered 55% of the area the on the radial topography, whereas only 12% of the area was covered by cells on the flat topography. To verify whether the radial topography could affect cellular function (e.g., proliferation and differentiation), migration-related signaling, and focal adhesion-related signaling, the expression of ERK and FAK was investigated [17, 28, 42]. The ERK and FAK protein levels were evaluated by western blotting. The results revealed upregulation of ERK and FAK in cells cultured on the radial patterned scaffolds compared to that in cells cultured on the flat patterned scaffolds (Fig. 4e).

Next, the secretion of growth factor from osteoblast like cells was analyzed from cells cultured on the radially topography. The osteoblast like cells on the radially topography secreted higher levels of growth factors such as basic fibroblast growth factor (bFGF), bone morphogenetic protein-4 (BMP-4), bone morphogenetic protein-7 (BMP-7), epidermal growth factor (EGF), epidermal growth factor receptor (EGFR), fibroblast growth factor-4 (FGF-4), fibroblast growth factor-7 (FGF-7), glia cell-derived neurotrophic factor (GDNF), hepatocyte growth factor (HGF), osteoprotegerin (OPG), transforming growth factor alpha, beta1, and beta3 (TGF α, TGF β1 and TGF β3) and insulin-like growth factor 1(IGF-1) compared to flat topography (Fig. 4f).

## Discussion

An ideal construct for treating large critical-sized bone defects should provide environments that enable complex living systems to mimic the natural healing process, such as topographical cues, mechanical properties, and chemical molecules [1]. Many studies have attempted to treat large critical-sized bone defects; however, very few clinical applications are available due to the limitations of materials and low functionality of the platforms [11, 43–46]. For the development of successful clinical applications, an understanding and consideration of transplantable scaffolds is necessary. The transplantable scaffold should be designed to allow the control of the mechanical properties, architectures, structures, biodegradability, and biocompatibility of the scaffold to enable the functional regeneration of the tissues. Based on these key considerations for designing a transplantable scaffold, we developed a topographically defined transplantable PCL-based scaffold to treat critical-sized bone defects considering three major factors, i.e., (i) topographical cues, (ii) suitable mechanical properties, and (iii) biodegradability and biocompatibility.

(i) Topographical cues: In an effort to develop tissue engineered biomaterials for bone regeneration, many research groups have investigated the interactions between host cells and the microenvironment of the bone tissue [30, 35, 47]. Surface topographical cues are thought to directly influence cell behavior of the bone-forming cells through intracellular signaling pathways mediated by focal adhesions. In particular, distinctively defined topographical substrates have been demonstrated to promote osteoblastic differentiation of osteoblasts without using external soluble chemical factors that could induce unwanted side effects. Additionally, wound healing is a crucial factor in the treatment of critical-sized bone defects for complete regeneration and the rate of early wound healing contributes significantly to successful bone regeneration. In general, wound healing is governed by two distinct cell behaviors, migration, and proliferation. Our results demonstrated a notable influence of radial topography in the migration and proliferation. First, the radial topographical cues significantly promoted the proliferation and migration of cells into the wound area both in vitro and in vivo [47]. Second, orientation of the radially pattern toward the center induced host-cell recruitment and cell-cell interactions. Third, the micro-topographically defined radially pattern provided an efficient environment for constructing bone tissue in which new osteoblasts and host cells from adjacent bone were recruited. Through the promotion of proliferation and migration, the cells migrated to the center of the defect site along the radial direction. We also investigated whether radial topography could affect molecular signaling. We found that radial topography affected ERK and FAK signaling, which are involved in cell function-related signaling pathways, such as proliferation, migration, and differentiation, and focal adhesion-related signaling pathways. Namely, the expression of ERK and FAK can be controlled by topographical cues to induce changes in cell morphology through cell-substrate interactions and to enhance cell proliferation and migration [48]. Furthermore, we confirmed that the topographical cue enhances the secretion of growth factors from osteoblast-like cells [49]. Specifically, the secretion of growth factors related to bone regeneration such as bFGF which induces calcium deposition [50], BMP-4 which induces bone healing and mineralization [51, 52], BMP-7 which increases ECM production [49], TGFβ which enhances osteogenic differentiation and bone marrow formation [53, 54] and IGF-1 which increases metabolic activity and enhances osteogenic differentiation was promoted [55].

(ii) Suitable mechanical properties: The mechanical properties of engineered scaffolds should match the mechanical properties of the tissue to be implanted as the scaffold should sufficiently support the tissue during tissue regeneration and block harmful external substances [56–59]. Bone tissue in particular is a sophisticated composite of different hierarchical structures, consisting of macrostructure, microstructure, and nanostructure. It exhibits the mechanical strength from several tens of MPa to strong mechanical strength (> 1 GPa) according to the hierarchical structure of bone tissue [60]. Considering the mechanical properties of the bone tissue, it is necessary to select appropriate biomaterials, having suitable mechanical strengths, for designing efficient scaffolds for BTE [15, 61–63]. The PCL polymer we used in the current study is one of the major synthetic polymers used for fabrication of scaffolds in the biomedical field owing to its good mechanical strength, extremely high elongation potential, and suitable flexibility. In addition, interestingly, it was confirmed that the mechanical properties of the PCL scaffolds were greatly improved by imprinting radial topography on the surface of the scaffold (Fig. 1(c-e). Our fabricated radial topographically defined scaffolds exhibited higher mechanical properties comparted to the previous flat and aligned topographically defined scaffolds. Specifically, our scaffolds showed more than twice the mechanical strength of the flat scaffold. Also, the one of the crucial factor to be considered in the mechanical properties of the scaffold is the scaffold stiffness. There have been many previous reports that different scaffold stiffness regulates cellular behaviors and bone regeneration [64–67]. Specifically, the moderately high stiffness of the scaffold promoted cell migration and bone differentiation, as well as bone regeneration in vivo rat bone defect model [66, 67]. In this respect, our radially patterned scaffold improved scaffold stiffness compared to previous PCL-based scaffolds, which promoted the cellular behavior and bone regeneration.

(iii) Biodegradability and biocompatibility: PCL is one of the most widely used polymers for biomedical applications owing to its advantageous properties, such as biocompatibility, high rigidity, and flexibility, and its controllable biodegradability within one to several years, depending on the molecular weight, degree of crystallinity of the polymer, and degradation conditions [68–70]. In addition, PCL has the capacity to promote the formation and growth of new tissues; further, it degrades during the tissue regeneration, while supporting and positioning the defects. This makes PCL a suitable material for replacing or treating damaged tissues. Moreover, PCL has already been approved by the US FDA and is relatively inexpensive, which means scaffolds based on this polymer can be more easily commercialized than those based on other biomaterials [68–70]. Based on these reasons, PCL is considered to be an ideal candidate for the development of scaffolds for bone regeneration [17]. In the current study, we chose PCL polymer for designing our transplantable scaffolds. Although PCL is a biocompatible and biodegradability material, it has poor bioactivity and reduced cell affinity, and it inhibits cellular interactions, a phenomenon that leads to low tissue regeneration rates (54). This limitation of synthetic polymers, including PCL, needs to be overcome if synthetic polymers are to be used for the development of efficient scaffolds for tissue engineering. To overcome the low bioactivity of PCL, we fabricated a radial topographical surface using lithography technology to improve its bioactivity and affinity with cells. Radial topography can improve the bioactivity of PCL by altering cell morphology and promoting cell migration [35].

Finally, we propose other possible applications and future plans for topographically defined PCL-based scaffolds. First, we propose a significant progression in the clinical application of biomaterial based BTE. For example, the relatively simple technology of imprinting topographical changes on the PCL surface was a useful strategy for the design and manipulation of the topographical structure as a potential scaffold for clinical use in critical-sized bone regeneration. Second, we propose an approach for further improving the performance of bone tissue regeneration based on our scaffolds. We can simply add some bioactive agents such as materials (e.g., collagen, gelatin, graphene, etc.), bone tissue regeneration-induced drugs or genes on the radially patterned PCL scaffolds; the synergic effects of radially patterned topographical cues and chemical cues on bone tissue regeneration would be expected. Third, we propose that topographically defined scaffolds can be used in a variety of applications for other tissues that consist of micro-topographical structures, such as cartilage, skin, muscle, and heart tissue. Although further study is needed regarding the clinical application of topographically defined PCL-based scaffolds for critical-sized bone regeneration, such as verification of its effectiveness in a large animal model, we have demonstrated the possibility of clinical application of topographically defined PCL-based scaffold.

## Data Availability

The datasets used and/or analyzed during the current study are available from the corresponding author on reasonable request.

## Abbreviations

PCL: Polycaprolactone
BTE: Bone tissue engineering
PLGA: Poly-lactic-co-glycolic acid
PGA: Poly-glycolic acid
PLA: Polylactic acid
ECM: Extracellular matrix
PUA: Polyurethane acrylate
ALP: Alkaline phosphatase
ARS: Alizarin red s
μ-CT: Microcomputed tomography
CEF: Cell elongation factor
CSI: Cell shape index
TCPS: Tissue culture polystyrene substrate
